# Partitioning the drivers of Antarctic glacier mass balance (2003–2020) using satellite observations and a regional climate model

**DOI:** 10.1073/pnas.2322622121

**Published:** 2024-09-30

**Authors:** Byeong-Hoon Kim, Ki-Weon Seo, Choon-Ki Lee, Jae-Seung Kim, Won Sang Lee, Emilia Kyung Jin, Michiel van den Broeke

**Affiliations:** ^a^Division of Glacier & Earth Sciences, Korea Polar Research Institute, Incheon 21990, Republic of Korea; ^b^Department of Earth Science Education, Seoul National University, Seoul 08826, Republic of Korea; ^c^Institute for Marine and Atmospheric Research, Utrecht University, Utrecht 3854 CS, The Netherlands

**Keywords:** Antarctica, ice mass change, surface mass balance, ice discharge

## Abstract

We investigate the mass changes of Antarctic glaciers from 2003 to 2020, partitioning them into the contributions of surface mass balance (SMB) and ice discharge, using high-resolution ice mass change estimates derived from the combination of two different types of satellite observations (gravimetry and altimetry) and outputs from a regional climate model. Our analysis indicates that changes in ice discharge have played a dominant role in ongoing ice mass trends and their accelerations, especially in glaciers near the Amundsen and Bellingshausen Seas in West Antarctica. In particular, mass losses of the Thwaites and Pine Island Glaciers have been mostly (>90%) controlled by ice discharge, while the contribution of SMB has been relatively minor. In East Antarctica, SMB accounts for significant portions (>50%) of ice mass imbalances of glaciers in e.g., Dronning Maud Land and Wilkes Land. Ice discharge has also played a notable role in overall mass gain in the region. While our ice discharge estimates agree well with previous estimates from satellite imagery in West Antarctica, notable differences are found in glaciers of East Antarctica and the Antarctic Peninsula. This highlights the need for more observations and improved numerical models to refine these estimates.

## Significance

Traditionally, understanding small-scale (like individual glaciers) mass changes in Antarctica has primarily relied on the flux gate method based on satellite imagery. This study advances our understanding of mass balance processes by combining two independent satellite observations measuring gravity and surface elevation changes to assess Antarctic glacier-scale ice mass changes from 2003 to 2020. We examined the contributing factors, namely the mass gains/losses from snowfall and ice discharge. Our results indicate increased ice discharge as the main factor in long-term ice mass change, albeit with significant regional variations. Comparisons of our estimates to those from satellite imagery reveal agreement in West Antarctica but notable regional differences, underscoring the importance of collective efforts to improve data coverage and model accuracy.

The Antarctic Ice Sheet (AIS) has been losing ice over recent decades, contributing significantly to global sea level rise. From 1992 to 2020, the AIS lost on average about 92 Gt y^−1^ (giga-tons per year) of ice, leading to a 7.4 mm increase in global mean sea level (1). With ongoing global climate warming, the Antarctic ice mass loss is expected to continue and even accelerate, potentially causing sea levels to rise by up to 0.34 m by the end of this century (2). Predicting these changes, however, is challenging due to the complexity of atmosphere–ocean–ice sheet interactions and the limitations of observational data (3). Improving the observational capability and deepening our understanding of the factors influencing ice mass variations are therefore essential for more accurate predictions of future sea level rise.

Two main processes are responsible for changes in ice mass: variations in surface mass balance (SMB) and/or in solid ice discharge. A third process, basal melt, is a minor contributor to the overall mass balance of grounded ice. SMB represents ice mass accumulation and ablation driven by atmospheric factors, while ice discharge is influenced by changes in ice flow speed and ice thickness at the grounding line. The main factor affecting SMB of the AIS are variations in snowfall (4), which take place on subannual, interannual, or longer (decadal) time scales (5). Antarctic ice discharge affects the mass change largely on interannual and longer time scales and is determined by factors such as glacier geometry, physical properties of the ice, and interactions between ice and bedrock or ocean (6–8). For each glacier basin, an imbalance between SMB and ice discharge results in either ice mass loss or gain. For instance, if warm ocean water causes an ice shelf to thin or disintegrate, this can reduce buttressing of the upstream glacier flow, possibly leading to increased ice discharge (9). This process has been observed recently in glaciers in West Antarctica and the Antarctic Peninsula (10–12). In addition, an increase/decrease in snowfall can increase/decrease glacier mass, as recently observed in, e.g., Dronning Maud Land in East Antarctica (13) and in coastal West Antarctica (14). These nonuniform ice mass changes make future predictions more difficult, underscoring the importance of separately determining SMB and ice discharge contributions (1).

AIS mass changes have been observed primarily using three remote sensing techniques: satellite gravimetry (GRACE), satellite altimetry, and a component method based on satellite imagery and estimates of SMB. Despite the differences in their sensitivity to various sources of uncertainty, these methods are employed independently by various research groups to achieve the most precise determination of AIS ice mass variations. The modern flux gate method, also known as the Mass Budget Method, Input–Output Method (1) or the Component Method (15), estimates ice mass change using ice discharge observations and modeled SMB. In this method, ice discharge is calculated by multiplying ice thickness with ice speed along a flux gate, typically located at or slightly upstream of the grounding line. Thanks to the availability of high-resolution glacier flow speed data, this method has been widely used to study glacier-scale ice mass balance (15). Yet, the flux gate method has its challenges. For example, in regions with complex bedrock topography, such as coastal East Antarctica and the Antarctic Peninsula, ice thickness measurements at the flux gates are often uncertain, as are SMB estimates from coarse atmospheric models (15, 16). In such cases, scaling ice discharge with limited observations across the entire basin is required, potentially introducing significant errors. Additionally, uncertainties in flow speed data would present another issue for this method. While these data are generally reliable for fast-moving glaciers, such as Thwaites Glacier in West Antarctica, the uncertainties become proportionally high for slower-moving glaciers (e.g., many in East Antarctica) (17), potentially affecting the accuracy of the results.

Efforts have also been made to investigate glacier-scale mass balance using GRACE (18, 19), which observes gravity potential changes inferred from range rate perturbation between two satellites. However, these estimates are hampered by signal leakages from land to oceans or from one basin to another due to the low spatial resolution (~300 km). Uncertainty in the correction for solid earth isostatic adjustment also introduces significant uncertainties into gravity-based ice mass change estimates (1, 20). Satellite altimetry, which measures the elevation change of the ice sheet surface, struggles with detecting glacier-scale ice mass variations in areas with complex ice topography, particularly in the Antarctic Peninsula. Converting observed changes in surface elevation to ice mass changes also introduces significant uncertainties, primarily due to the variability in firn densification (21).

To combine the strengths and reduce the limitations of GRACE and altimetry, a recent study (22) developed a novel method that combines both methods to improve the spatial resolution of GRACE data and reduce the uncertainty of altimetry data. This results in high spatial resolution ice mass variations comparable to that of firn-corrected altimetry data (~27 km), and its accuracy is similar to that of GRACE. The estimates with higher accuracy and spatial resolution along with SMB data from a regional climate model allow us to estimate glacier scale ice mass changes and investigate their primary source. In this study, we estimate AIS mass change during 2003–2020 using the methodology developed in the aforementioned study, with the inclusion of a new satellite mission (*Materials and Methods*). We evaluate the relative contributions of the two primary processes, SMB and ice discharge, in determining ice mass trends and accelerations. Various sources of uncertainty affecting the linear trend and acceleration terms are addressed to ascertain the robustness of our results. Finally, we compare our ice discharge estimates with those derived from satellite imagery and ice thickness.

## Results

### Entire AIS.

**Fig. 1.**
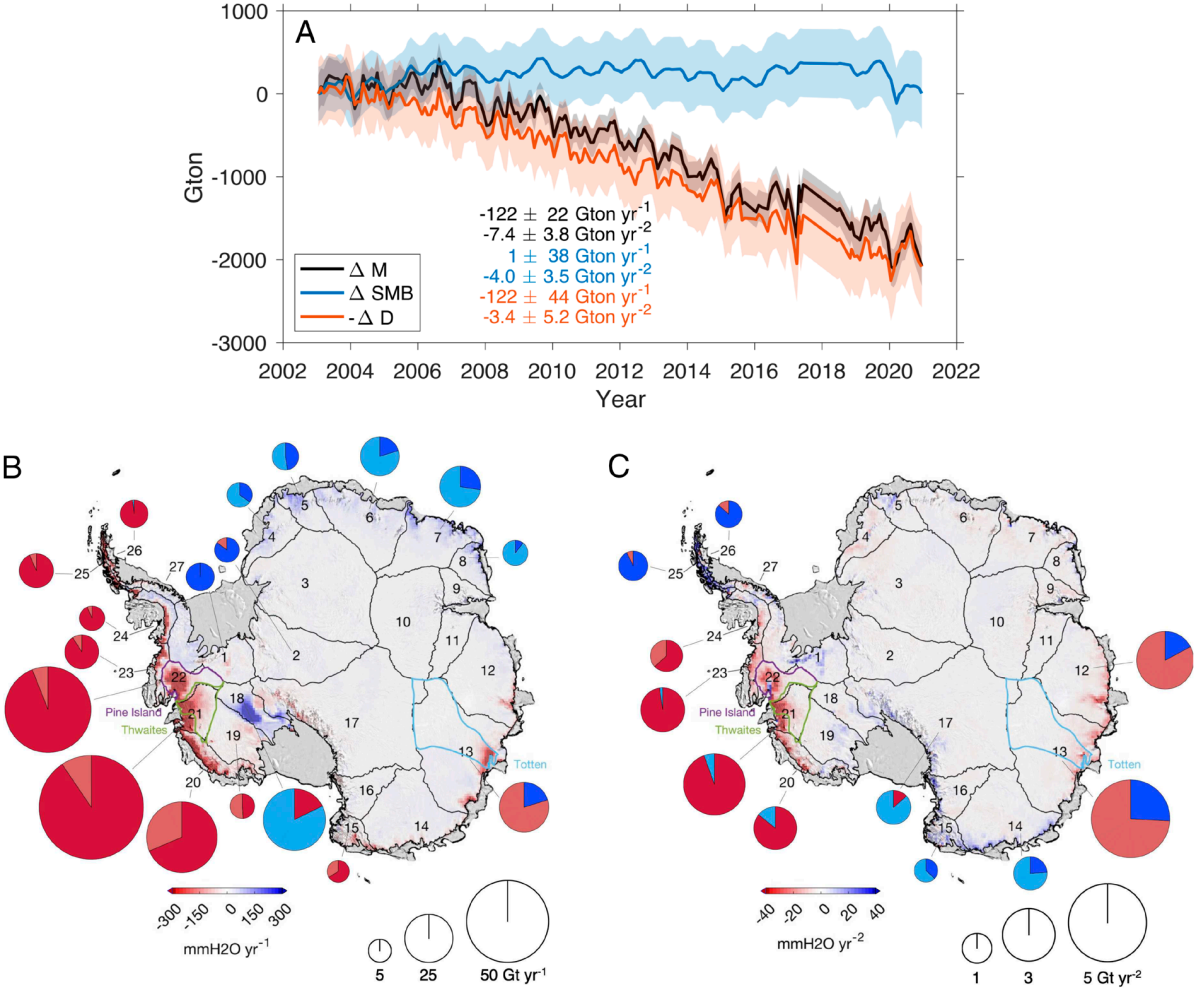
(*A*) ΔM, ΔSMB, and -ΔD over AIS. Note that ΔSMB and -ΔD were resampled to the time intervals of ΔM (GRACE observation). The ΔD is presented with its negative sign (-ΔD) for a more intuitive interpretation. (*B* and *C*) Maps of linear trends (*B*) and accelerations (*C*) in ΔM, accompanied by pie charts showing the contribution of ΔSMB (light colors) and -ΔD (dark colors). Circle sizes indicate the combined absolute values of SMB and ice discharge, represented by blue (positive values) and red (negative values) colors, respectively. Only statistically significant values of ΔM (Table 1) are displayed as pie charts. The black lines represent the basin boundaries from ICESat altimetry observation (23). The boundaries of the Pine Island (purple), Thwaites (green), and Totten (cyan) Glaciers were obtained from satellite radar imagery (24, 25). The background shading represents the optical image from the MODIS Mosaic of Antarctica 2013–2014 (MOA2014) project (26).

From 2003 to 2020, the estimated ice mass change (ΔM) over the entire AIS shows significant mass loss at a rate of −122 ± 22 Gt y^−1^ with an acceleration of −7.4 ± 3.8 Gt y^−2^ (black lines, Fig. 1*A*). This resulted in an 18-year total loss of about 2100 Gt, which corresponds to a global mean sea level rise of 5.8 mm.

The spatial patterns of linear trends (Fig. 1*B*) and accelerations (Fig. 1*C*) vary across the AIS. As noted in previous studies, glaciers in West Antarctica and the Antarctic Peninsula (basins 20 to 26) adjacent to the Amundsen Sea and Bellingshausen Sea, as well as the Totten Glacier in East Antarctica (basin 13), have exhibited notable mass losses. On the other hand, the stagnant Kamb Ice Stream (basin 18) in West Antarctica and glaciers in coastal Dronning Maud Land (basins 4 to 8) in East Antarctica have exhibited mass gains. Mass loss accelerations are noted for the Pine Island (basin 22) and Thwaites Glaciers (basin 21) in West Antarctica, as well as for glaciers in the western parts of Wilkes Land in East Antarctica (basins 12, 13). On the other hand, the eastern parts of Wilkes Land (basins 14, 15) show glaciers with notable positive mass change accelerations. This suggests that the overall AIS ice mass loss and acceleration observed in Fig. 1*A* result from spatially variable changes across the AIS.

The ice mass change, ΔM, is determined by the imbalance between time-integrated SMB rate (∫SMB dt) and ice discharge rate (∫D dt). Accordingly, the contribution of ice discharge to ΔM can be isolated by subtracting ∫SMB from the ΔM if the latter is known from e.g., gravimetry (5, 19). To partition contributions from SMB and ice discharge to ΔM, a reference SMB value during a standard period must be defined (*Materials and Methods*). Similar to the methodology of previous studies (15, 27–29), we used the mean SMB from 1979 to 2008 as the reference value ($SMB_{\mathrm{ref}}$). Cumulative anomalies in time-integrated SMB relative to the reference value (ΔSMB) can be obtained and finally used to estimate ΔD (ΔM-ΔSMB). This method contrasts with the flux gate method (e.g., ref. 15), which calculates ΔM by explicitly adding ∫SMB dt to the observed ∫D dt. For SMB, we used the Regional Atmospheric Climate Model (RACMO) version 2.3p2 (30) forced at the lateral boundaries by the European Centre for Medium-Range Weather Forecasts (ECMWF) reanalysis ERA5, which has been thoroughly evaluated using in situ SMB observations and near-surface climate data (31) (*Materials and Methods*).

The blue and red lines in Fig. 1*A* represent ΔSMB and -ΔD, along with their associated uncertainties (*Materials and Methods*). The similar trends of ΔM and -ΔD suggest that increased ice discharge has prompted an overall mass loss throughout Antarctica. The estimated trend in -ΔD is −122 ± 44 Gt y^−1^, accounting for >~100% of the ΔM trend (about −122 Gt y^−1^). On the other hand, no clear trend is found in ΔSMB during the same period, suggesting that the mean value of SMB over the past 20 y has remained similar to the reference period.

However, ΔSMB exhibits a statistically significant acceleration in loss over the AIS at −4.0 ± 3.5 Gt y^−2^. Although this indicates a decrease in SMB trend during 2003–2020, it is important to note that the apparent statistical significance is primarily due to a couple of lower SMB years at the end of the time series, which might reflect interannual variability rather than a true acceleration in loss. This apparent acceleration of ΔSMB accounts for roughly 54% of ΔM ‘s acceleration, marking it as a key contributor to the ice mass loss acceleration. The remaining 46% has been attributed to -ΔD, but the value is not significant considering its CI (−3.4 ± 5.2 Gt y^−2^).

### Mass Balances by Basins.

The AIS is divided into 27 drainage basins according to surface elevations observed by ICESat satellite altimetry (23) (Fig. 1 *B* and *C*). These 27 basins have been used in previous studies to investigate regional ice mass balance (1, 19). We calculated ΔM for these 27 basins and separated the contribution of ΔSMB and -ΔD. The individual time series for each basin are shown in *SI Appendix*, Fig. S2, and a summary of their linear trends and accelerations, along with their CI, is provided in Table 1. Of the 27 basins, 18 basins (basins 1, 2, 4 to 8, 13, 15, and 18 to 26) show significant trends in ΔM. Regarding mass change accelerations, only 11 basins (basins 12 to 15, 17, 20 to 23, 25, and 26) exhibit statistically significant values.

**Table 1.** Linear trends and accelerations of Δ*M*, Δ*SMB*, and -Δ*D* over 27 Antarctic drainage basins

	Linear trend (Gt y^−1^)			Acceleration (Gt y^−2^)		
Basin number	Δ*M*	Δ*SMB*	−Δ*D*	−Δ*M*	Δ*SMB*	−Δ*D*
1	**8.9 ± 8.7**	1.0 ± 3.1	7.9 ± 9.3	0.6 ± 0.7	0.5 ± 0.8	0.1 ± 1.0
2	**4.2 ± 3.4**	−0.7 ± 1.1	**4.9 ± 3.6**	0.1 ± 0.4	**0.3 ± 0.2**	−0.2 ± 0.5
3	8.7 ± 9.3	**6.8 ± 1.8**	1.9 ± 9.4	−0.4 ± 0.4	**−0.9 ± 0.4**	0.5 ± 0.6
4	**5.5 ± 3.2**	**3.3 ± 1.4**	2.2 ± 3.5	−0.5 ± 0.5	−0.3 ± 0.4	−0.2 ± 0.7
5	**7.0 ± 1.9**	**3.3 ± 1.6**	**3.7 ± 2.5**	0.5 ± 0.6	0.4 ± 0.5	0.1 ± 0.7
6	**14.1 ± 4.1**	**9.6 ± 4.2**	4.5 ± 5.9	0.0 ± 1.2	0.6 ± 1.4	−0.6 ± 1.9
7	**16.0 ± 4.3**	**11.5 ± 2.7**	4.5 ± 5.1	−0.4 ± 0.9	−0.3 ± 0.8	−0.1 ± 1.2
8	**6.3 ± 1.6**	**5.4 ± 1.0**	0.9 ± 1.9	0.0 ± 0.3	−0.2 ± 0.3	0.2 ± 0.4
9	0.6 ± 1.3	**1.0 ± 0.5**	−0.4 ± 1.4	−0.2 ± 0.2	**−0.4 ± 0.1**	0.1 ± 0.2
10	3.3 ± 4.7	0.1 ± 1.0	3.2 ± 4.8	0.4 ± 0.4	**−0.7 ± 0.2**	**1.2 ± 0.4**
11	0.3 ± 1.8	0.3 ± 0.5	0.0 ± 1.8	−0.1 ± 0.2	−0.1 ± 0.1	0.0 ± 0.3
12	−0.9 ± 3.6	**−13.2 ± 3.4**	**12.2 ± 5.0**	**−2.1 ± 0.5**	**−2.6 ± 0.6**	0.5 ± 0.8
13	**−17.0 ± 2.3**	**−22.8 ± 5.3**	**5.8 ± 5.7**	**−2.4 ± 0.8**	**−3.6 ± 0.9**	1.2 ± 1.2
14	3.9 ± 3.9	**−6.0 ± 3.7**	**9.9 ± 5.4**	**1.1 ± 0.9**	**1.1 ± 0.9**	0.0 ± 1.3
15	**−3.9 ± 1.6**	**−1.2 ± 0.8**	**−2.8 ± 1.8**	**0.6 ± 0.3**	**0.3 ± 0.2**	0.3 ± 0.4
16	0.2 ± 1.4	0.4 ± 0.4	−0.3 ± 1.4	0.2 ± 0.2	**0.2 ± 0.1**	0.0 ± 0.3
17	2.7 ± 5.3	−1.2 ± 2.1	4.0 ± 5.8	**0.9 ± 0.5**	**1.2 ± 0.5**	−0.2 ± 0.7
18	**22.6 ± 1.9**	**28.6 ± 0.5**	**−6.0 ± 2.0**	0.1 ± 0.2	0.1 ± 0.2	0.0 ± 0.3
19	**−5.5 ± 2.9**	**−3.0 ± 1.2**	−2.5 ± 3.2	0.4 ± 0.5	−0.1 ± 0.3	0.4 ± 0.6
20	**−41.0 ± 3.6**	**−11.9 ± 3.3**	**−29.0 ± 4.9**	**−1.5 ± 1.2**	0.3 ± 0.9	**−1.8 ± 1.5**
21	**−69.3 ± 3.6**	**−5.7 ± 2.8**	**−63.6 ± 4.5**	**−2.8 ± 1.0**	0.3 ± 0.7	**−3.1 ± 1.2**
22	**−51.4 ± 3.9**	−2.0 ± 2.6	**−49.5 ± 4.7**	**−2.0 ± 1.1**	0.3 ± 0.8	**−2.2 ± 1.4**
23	**−12.2 ± 2.5**	−1.2 ± 1.6	**−11.0 ± 3.0**	**−1.0 ± 0.6**	−0.3 ± 0.4	−0.7 ± 0.7
24	**−6.0 ± 2.5**	0.0 ± 2.3	**−6.0 ± 3.4**	−0.2 ± 0.8	0.2 ± 0.7	−0.4 ± 1.0
25	**−11.5 ± 3.2**	−0.9 ± 1.5	**−10.6 ± 3.6**	**0.8 ± 0.4**	−0.2 ± 0.3	**1.0 ± 0.5**
26	**−7.6 ± 2.1**	−0.1 ± 0.8	**−7.5 ± 2.2**	**0.6 ± 0.3**	−0.2 ± 0.2	**0.7 ± 0.4**
27	0.6 ± 1.6	**−0.7 ± 0.5**	1.4 ± 1.7	0.0 ± 0.3	0.1 ± 0.1	−0.1 ± 0.3
WA	**−148.0 ± 19.6**	5.9 ± 14.4	**−153.8 ± 24.3**	**−6.2 ± 4.4**	1.2 ± 3.5	**−7.4 ± 5.6**
EA	**50.9 ± 25.5**	−3.4 ± 24.7	**54.3 ± 35.5**	−2.3 ± 3.7	**−5.0 ± 4.2**	2.7 ± 5.6
AP	**−24.4 ± 5.8**	−1.6 ± 4.6	**−22.8 ± 7.4**	1.2 ± 1.4	−0.1 ± 1.0	1.3 ± 1.7
AIS	**−121.5 ± 21.6**	0.8 ± 38.2	**−122.3 ± 43.9**	**−7.4 ± 3.8**	**−4.0 ± 3.5**	−3.4 ± 5.2

Statistically significant values are highlighted in bold texts.

#### West Antarctica.

In West Antarctica (basins 1, 18 to 23), substantial mass loss is observed for glaciers terminating in the Amundsen and Bellingshausen Seas (basins 20 to 23), supporting findings from earlier studies (1, 32). The mass loss rate over basins 20 to 23 is −173.9 ± 6.9 Gt y^−1^, about 1.5 times greater than the total mass loss rate of the entire AIS. The main driver of this mass loss is -ΔD, with regionally varying contributions. In basins 21 to 23, -ΔD accounts for 90 to 101% of the mass loss. However, in basin 20, which includes glaciers flowing into the Getz Ice Shelf, there has been a non-negligible contribution from ΔSMB (~29%), in agreement with a previous study (27).

Basin 19, which includes Bindschadler, MacAyeal, and Echelmeyer Ice Streams, exhibits a mass loss of −5.5 Gt y^−1^. ΔSMB is responsible for about 54% of the mass loss, and -ΔD accounts for the rest, although it is not statistically significant.

In Basin 18, encompassing the Kamb Ice Stream and the northern tributary of the Whillans Ice Stream, mass changes are largely governed by SMB due to the stagnant nature of Kamb Ice Stream. Nominally, -ΔD in the basin is thought to be zero, and thus the SMB trend is calculated using ∫SMB dt instead of ΔSMB. The linear trends of ΔM and ∫SMB dt are about 22.6 Gt y^−1^ and 28.6 Gt y^−1^, respectively, leaving a significant trend in -ΔD (about −6.0 ± 2.0 Gt y^−1^). Nevertheless, when we employ a more accurately constrained basin boundary for the Kamb Ice Stream, we find that ΔM and ∫SMB dt are nearly identical, about 23.7 Gt y^−1^ and 24.5 Gt y^−1^, respectively, which yields -ΔD close to zero (*SI Appendix*, Fig. S3), validating our approach.

In total, glaciers in West Antarctica have been losing ice mass at a rate of −148.0 Gt y^−1^, with an acceleration in loss of −6.2 Gt y^−2^. The main component of these long-term variations is -ΔD, accounting for about −153.8 Gt y^−1^ and −7.4 Gt y^−2^. The contributions from ΔSMB have been relatively minor.

#### Antarctic Peninsula.

Many glaciers in the Antarctic Peninsula have been retreating in recent decades (33, 34), mainly due to oceanic influences (10, 35). Our analysis of the four basins in the Antarctic Peninsula (basins 24 to 27) reveals pronounced mass loss in three basins (basins 24 to 26), and no trend in basin 27.

Basin 24, adjacent to the Bellingshausen Sea, exhibits a strong negative mass trend along the English Coast (Fig. 1*B*). Previous research based only on GRACE observations (34) also reported mass loss in this area, but it was combined with the mass loss of basin 23 in West Antarctica due to the low spatial resolution of GRACE data. Our study effectively assigns the mass loss to basin 24, which is approximately −6.0 Gt y^−1^. This loss is fully attributed to -ΔD (~100%).

Basins 25 and 26, located at the northern tip of the Antarctic Peninsula, have been losing ice mass at a rate of −19.1 Gt y^−1^. These losses are largely (~95%) triggered by changes in -ΔD. Interestingly, small but statistically significant decelerations in loss are observed in both ΔM and -ΔD, which differs from previous reports of persistent grounding line retreat and an increase in ice discharge of glaciers in the Antarctic Peninsula (15, 33). We attribute this to the temporary decrease in ice discharge in the late 2010s (basins 25 and 26 in *SI Appendix*, Fig. S2), potentially influenced by changes in ice shelf buttressing and, which interacts with bed geometry and generally varies interannually (36). However, further investigation is required, using data from ocean surveys and other remote sensing or in situ data, to validate these results.

In total, the ice mass loss of Antarctic Peninsula glaciers is estimated to be −24.4 Gt y^−1^, which represents about 20% of the mass loss of the entire AIS. Similar to West Antarctica, this mass loss is predominantly (~93%) induced by -ΔD. The mass loss acceleration is statistically negligible.

#### East Antarctica.

The trends in ΔM in East Antarctica (basin 2 to 17) display a dipole pattern with increasing ice mass in Dronning Maud Land and decreasing ice mass in Wilkes Land and the northern part of Victoria Land (Fig. 1*B*). Glaciers in Dronning Maud Land (basin 4 to 8), influenced by increased snowfall since 2009 (13), have gained ice mass at an average estimated rate of 48.8 Gt y^−1^. Our analysis indicates that the primary driver of this mass gain is ΔSMB, accounting for 53 to 90% of the increased ice mass. The rest of the ice mass increase can be attributed to -ΔD, but values for individual basins are statistically insignificant, except for basin 5. Earlier estimates using the flux gate method revealed a similar mass gain in some parts of Dronning Maud Land (Figure 2D in ref. 15), suggesting a potential slowdown of glacial flow in this region.

For basin 13 in Wilkes Land, the mass loss rate is estimated to be −17 Gt y^−1^, with an accelerating rate of loss (−2.4 Gt y^−2^). Our analysis suggests that the primary driver of this mass loss and acceleration in this region is ΔSMB. Basin 15 in Victoria Land also displays a mass loss of about −3.9 Gt y^−1^, but with a decelerating rate of loss (0.6 Gt y^−2^). ΔSMB accounts for about 30% of this loss and about 60% of the deceleration. Of the 16 basins in East Antarctica, only five (basins 12 to 15, and 17) show statistically significant accelerations in ΔM, contrasted by opposite signs: negative (acceleration in loss) in 12 and 13 and positive (acceleration in gain) in 14 and 17. These accelerations in ΔM are largely (>63%) linked to changes in ΔSMB.

In summary, the basins in East Antarctica have shown ice mass gain of 50.9 Gt y^−1^, with no significant acceleration. Regionally, while ΔSMB plays an important role in both trends and accelerations of ΔM, its overall contribution for East Antarctica is minor due to the cancellation of positive and negative contributions (Fig. 1*B*). On the other hand, while linear trends in -ΔD are relatively smaller than those of ΔSMB in each basin, when integrated, -ΔD explains nearly all (~100%) of ΔM over the entire East Antarctica.

### Glacier Scale Mass Balances.

Since our combined estimates for ΔM are of sufficiently high resolution (about 27 km), it is possible to distinguish mass changes even at finer scales, such as individual glaciers (22). Satellite imagery, detailing ice flow directions and speeds, has enabled us to define the boundaries for individual Antarctic glacier basins (24, 25). Using these data, we revisited three glaciers (Pine Island, Thwaites in West Antarctica, and Totten in East Antarctica) where substantial ice losses have been previously reported (37, 38). Note that the basin boundary of Pine Island Glacier is nearly identical to that of the previously delineated basin 22, whereas the boundaries of Thwaites and Totten Glaciers are smaller than those of basin 21 and 13, respectively, due to the exclusion of small, peripheral glacier catchments, which can be distinguished by our ice mass estimates. These sub-basin delineations are used to ensure comparability with prior estimates from the flux gate method (15), which will be addressed in detail in the next section.

**Fig. 2.**
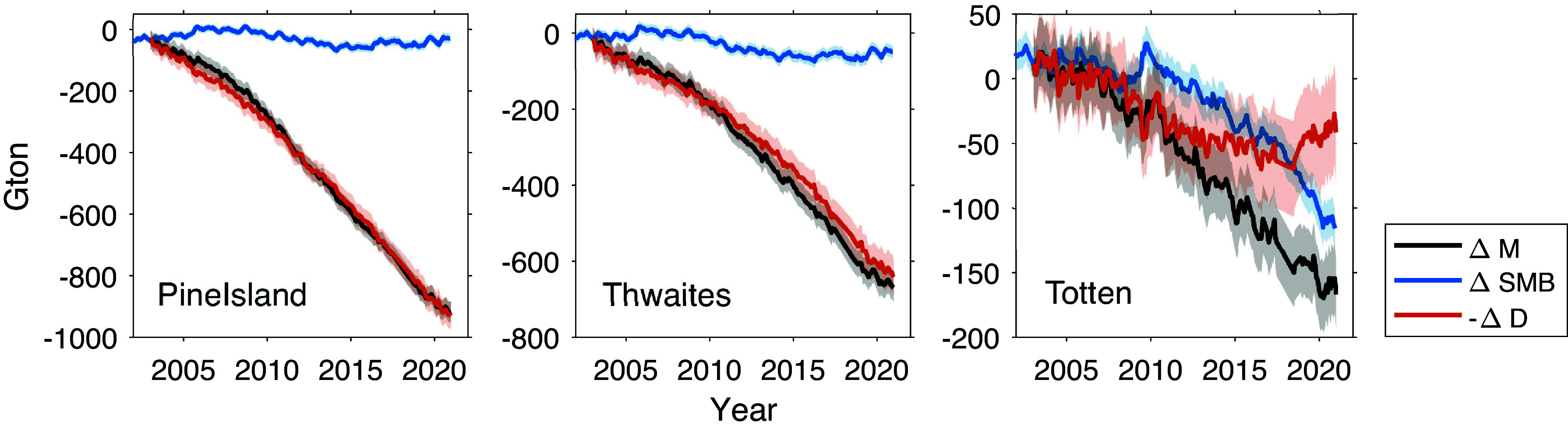
ΔM (black), ΔSMB (blue), and -ΔD (red) at the Pine Island (*Left*), Thwaites (*Middle*), and Totten (*Right*) Glaciers.

Pine Island and Thwaites Glaciers (outlined by purple and green lines in Fig. 1 *B* and *C*) have emerged as main contributors to AIS mass loss. From 2003 to 2020, these glaciers showed a marked decline in ΔM (black lines), with rates of −49.0 ± 1.2 Gt y^−1^ and −35.5 ± 2.1 Gt y^−1^ (Fig. 2 *A* and *B*). These rates account for 40% and 29% of the total AIS mass loss, respectively. Most of the mass loss, specifically 97% (−47.6 ± 2.6 Gt y^−1^) for Pine Island and 90% (−31.8 ± 3.1 Gt y^−1^) for Thwaites, is attributed to -ΔD (red lines). In addition, discernible accelerations in mass loss (−1.4 ± 1.0 Gt y^−2^ for Pine Island and −1.2 ± 0.6 Gt y^−2^ for Thwaites) are found when linear trends are subtracted from the time series (*SI Appendix*, Fig. S4). These accelerations, representing 36% of the total accelerated AIS mass loss, are also driven primarily by -ΔD (−1.6 ± 1.2 Gt y^−2^ for Pine Island and −1.6 ± 0.8 Gt y^−2^ for Thwaites).

For Totten Glacier (outlined by cyan lines in Fig. 1 *B* and *C*), ΔM decreased by −10.5 ± 0.9 Gt y^−1^, with an acceleration in loss of −0.5 ± 0.3 Gt y^−2^ (Fig. 2*C*). Unlike for Pine Island and Thwaites Glaciers, ΔSMB exhibits a greater influence on Totten Glacier and explains about 63% of the mass loss in this region, surpassing the contribution of -ΔD. A slight (0.6 ± 0.4 Gt y^−2^) deceleration in loss is found for -ΔD due to a slowdown in ice speed around mid-2010 (red line in Fig. 2*C*). In a previous study (37), the interannual variations in the grounding line dynamics of Totten Glacier, influenced by ocean temperature changes, can occasionally disrupt the glacier flow speeds. Our analysis from satellite imagery data further supports this, showing a 5% reduction in ice speeds near the grounding line since 2015 (*SI Appendix*, Fig. S5), which aligns well with previous estimates (39).

#### Comparison to Previous Estimates.

There are few studies available to compare our ice mass changes estimated at the basin and sub-basin scales (15, 39, 40). The most comprehensive is ref. 15, which provides 1979–2017 annual ice discharge rates (D) at all AIS drainage basins using the flux gate method. We compare two long-term components (linear trend and acceleration) of our indirectly derived ice discharge estimate with those directly obtained from the flux gate method. We also incorporate results from other studies (39, 40) for several other glaciers. For a comprehensive comparison of long-term components within basins, the linear trends and accelerations in -ΔD are estimated from each dataset during the period 2003–2017. We then normalized to specific mass balance (mass change per unit area) by dividing by each glacier’s area. For comparison between different datasets for -ΔD, we select glaciers that fulfill the following criteria:

1) Given the effective spatial resolution of our estimates, glaciers less than 2,000 km^2^ in extent (less than three pixels) were excluded (22).
2) Glaciers that the flux gate method assumes as having unchanged ice mass [where ice discharge is assigned an identical value to the SMB model (15)] were also omitted.
3) Glaciers lacking statistically significant linear trends or acceleration in our evaluations were excluded.

Following these criteria, we retain 40 glaciers (17 from West Antarctica, 19 from East Antarctica, and 4 from the Antarctic Peninsula) for our linear trend comparison and 29 glaciers (15 from West Antarctica, 10 from East Antarctica, and 4 from the Antarctic Peninsula) for our acceleration comparison. The selected glaciers encompass about 43% of the total area of the AIS for the linear trend comparison and 39% for the acceleration comparison. The resulting list of glaciers is provided in *SI Appendix*, Table S3.

**Fig. 3.**
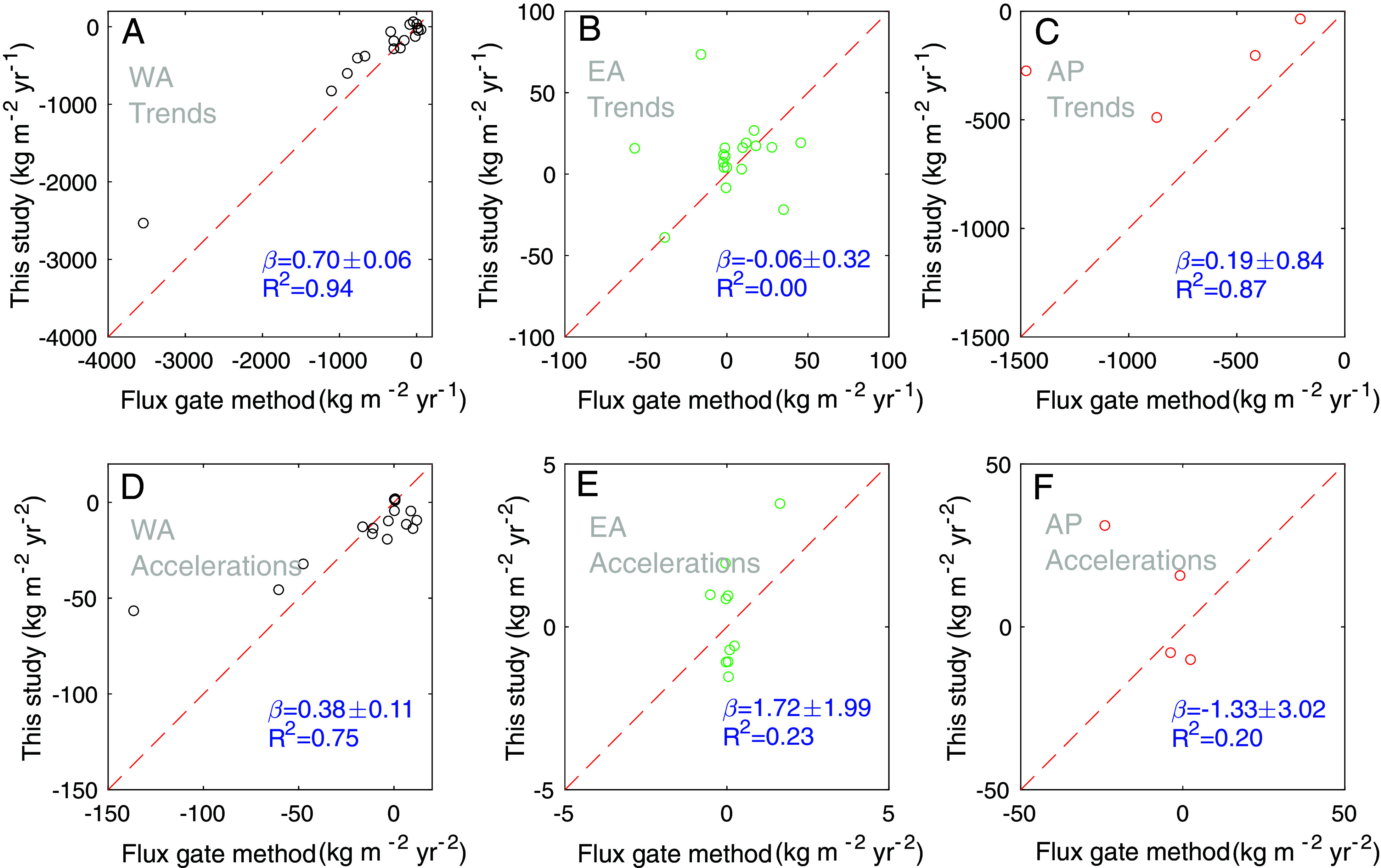
Comparison of trends (*A*–*C*) and accelerations (*D*–*F*) in ice discharge changes (-ΔD) estimated in this study with those from the flux gate method (15) across glaciers in the West Antarctica (*A* and *D*), East Antarctica (*B* and *E*), and Antarctic Peninsula (*C* and *F*). The refined list of glacier basins is provided in *SI Appendix*, Table S3.

We first assess the agreement between the long-term components of our discharge estimate and those of the flux gate method by quantifying the R-square (R^2^) and the regression coefficient (β) (Fig. 3 *A*–*F*). While we find a moderate agreement between the two estimates (β≈0.64 and R^2^ = 0.74 for linear trends, β≈0.41 and R^2^ = 0.56 for accelerations), the level of agreement varies regionally. For the glaciers in West Antarctica (Fig. 3 *A* and *D*), the two estimates generally display a high correlation (R^2^ = 0.94 for trend, R^2^ = 0.75 for acceleration). Nonetheless, our acceleration estimates are small in comparison to the flux gate method (β≈0.38). The most pronounced differences in acceleration of ice discharge are observed in the basin encompassing the Smith and Pope Glaciers. When these are excluded, the similarity between the two methods improves (β≈0.51).

There are marked discrepancies between the two methods in East Antarctica, where we find low R^2^ and small β values for both linear trends and accelerations (Fig. 3 *B* and *E*). Our results show statistically significant -ΔD contribution to ice mass increase for four adjacent glaciers in Dronning Maud Land (Jutulstraumen, Jelbart, Nivl, and Vigrid) (*SI Appendix*, Fig. S6 *A–D*), while the flux gate method shows values close to zero (39, 40). Additionally, our -ΔD estimate shows the similar effect to ice mass increase for Denman Glacier in Wilkes Land (*SI Appendix*, Fig. S6*E*) associated with ice discharge decrease, which contrasts with the gradual increase in ice discharge observed since the 1970s from the flux gate method (15, 41). For acceleration, the flux gate method generally yields near-zero values, while our estimates present a broader range (Fig. 3*E*). However, similarity is shown in some glaciers, such as Denman, which commonly yields deceleration in loss of -ΔD and is supported by an independent study (39).

For glaciers in the Antarctic Peninsula, specifically those flowing into the George VI, Stange, and Larsen C Ice Shelves, and those in West Graham Land, our estimates show strong correlations (R^2^ = 0.87) in trends with those from the flux gate method (Fig. 3*C*), but the values are much smaller (β≈0.19). In terms of accelerations (Fig. 3*F*), we observed a low correlation (R^2^ = 0.20) and similarity (β≈-1.33) between the two datasets. However, this comparison is limited by the small number of glaciers included at the AP (4 glaciers, covering 55% of the Antarctic Peninsula area). Other glaciers were excluded from our analysis due to their small sizes (11 glaciers), the presupposed state of balance (27 glaciers), or statistically negligible values (5 glaciers).

## Discussion

Previous studies have highlighted significant disagreements in long-term ice mass change estimates between the flux gate method and GRACE (or altimetry). The ΔM loss from the flux gate method over AIS was about 100 Gt y^−1^ larger than other estimates, with substantial contributions from East Antarctica (1, 42). Similarly, our indirect -ΔD estimates for smaller basins also showed significant discrepancies when compared to the flux gate method, particularly in East Antarctica and the Antarctic Peninsula. Part of such discrepancies is likely due to underestimation by GRACE in detecting ice mass loss. For example, improving the accuracy of mantle viscosity representation in GIA models may increase the total AIS mass change estimates by up to 20 Gt y^−1^ (43), which accounts for about 20% of the overall ΔM discrepancy. The GIA model errors inferred by the Global Navigation Satellite System (GNSS) also suggested uncertainties in ΔM on the order of 10 to 15 Gt y^−1^ (20). The GIA uncertainties in West Antarctica were corrected here (*Materials and Methods*) but would remain for the rest of AIS.

The discrepancy in ΔM can also be partly attributed to uncertainties in the flux gate method. The flux gate method directly computes ice discharge by multiplying observed ice speeds with ice thickness estimates at the grounding line. However, comprehensive estimates of ice discharge from satellite imagery in Antarctica (15) suggest that a significant portion of ice thickness and ice speed at grounding lines remain poorly constrained. In regions where the bed topography is not well observed, ice thickness derived from ice shelf freeboard observations using satellite altimetry is inaccurate. Furthermore, in glaciers particularly in the Antarctic Peninsula and East Antarctica, where there is lack of ice thickness observations at groundling lines, ice discharge was estimated from scaled SMB relative to the value during the reference period. Additionally, the ice speed observations used in the flux gate method sometimes scale maximum speeds at the flux gate rather than using the full speed profile passing through the gate. These incomplete observations can lead to systematic errors in the ice discharge estimates, significantly affecting the estimation of linear trends. The ice speed data themselves also contain inherent errors due to the spatial resolution of satellite images, typically about tens of meter per year (17), which makes it insensitive to capturing subtle changes in ice speed where flow speed of the glacier is small. Moreover, missing data due to instrumental issues and uncertainties in ice speed estimates necessitate time interpolation (15), which can introduce errors into both linear trend and acceleration of ice discharge (e.g., *SI Appendix*, Fig. S7).

Potential long-term errors in the SMB model (in our study RACMO forced by ECMWF reanalysis) would impact both our calculation of -ΔD from observed ΔM and the flux gate method’s calculation of ΔM by adding SMB to ice discharge estimates. We investigated the sensitivity of our results to the choice of SMB model by comparing different SMB products: three reanalysis fields (i.e., ECMWF reanalysis 5 (ERA5) (44), The Modern-Era Retrospective analysis for Research and Applications Version 2 (MERRA2) (45), and Japanese 55-y Reanalysis (JRA55) (46)). This shows that the choice of reanalysis significantly affects the long-term components of regional ΔSMB (*SI Appendix*, Figs. S8 and S9). This discrepancy is particularly pronounced in the linear trends of ΔSMB in Dronning Maud Land (basins 4 to 8) and Wilkes Land (basins 12 and 13), with deviations reaching up to 25 Gt y^−1^ (*SI Appendix*, Fig. S8). For the mass change acceleration, most basins show small deviations within 1 Gt y^−2^, but basin 13 in Wilkes Land (where Totten Glaciers is located) shows a particularly large deviation of 4.6 Gt y^−2^ (*SI Appendix*, Fig. S9). However, caution is required as these differences might not be reflective of the actual uncertainty, as previous studies clearly indicate that ERA5 reanalysis best represents Antarctic SMB (47, 48). A comparison of RACMO ΔSMB with an independent regional climate model, Modèle Atmosphérique Régional (MAR), forced by the same ERA5 reanalysis (49), shows much smaller deviations in the linear trends (0.1 to 5.0 Gt y^−1^) and accelerations (0.0 to 0.2 Gt y^−2^) in Dronning Maud Land and Wilkes Land (*SI Appendix*, Fig. S10). This suggests that a significant portion of the ΔSMB differences listed above arise from the reanalysis fields rather than differences in the physics of the regional climate models. The potential errors in ΔSMB are currently challenging to quantify accurately and should be assessed through comprehensive, long-term comparisons with observed SMB data from widespread regions of Antarctica.

Considering these error factors, the cause of discrepancies in -ΔD between our estimates and the flux gate method shown in Fig. 3 can be partly explained. For example, the Smith and Pope Glaciers in West Antarctica show a complex spatial distribution of ice speeds near the grounding line, making the scaling method used in ref. 15 potentially invalid, as it can lead to an incomplete capture of the spatial variability in ice flow across the catchments (39). Inaccuracy in our estimates including various uncertainty sources from ΔM and ΔSMB, would partly explain the discrepancies between the two -ΔD estimates in East Antarctica. Consequently, while our estimates serve as an alternative for glaciers poorly constrained by the flux gate method, further improvements in the uncertainties of ΔM and ΔSMB are necessary for more accurate assessments.

## Conclusion

We used high-resolution ice mass change estimates based on combined satellite gravimetry and altimetry and a numerical SMB model to investigate the continental- to regional-scale ice mass balance in Antarctica over the past 20 years. The results show that the spatially nonuniform ice mass changes were due to different contributions from SMB and ice discharge. Increased ice discharge was found to be the primary cause of the significant ice mass loss and acceleration of, notably, the glaciers flowing into the Amundsen and Bellingshausen Seas, as well as the glaciers in the Antarctic Peninsula. In contrast, SMB variability mostly explains the ice mass gain in Dronning Maud Land, mass loss in Wilkes Land, and the accelerations in the eastern part of East Antarctica (basins 12 to 17). However, the spatially varying linear trends in SMB canceled out across the entire continent, making ice discharge the dominant contributor (~100%) to the ice mass loss. However, SMB did explain 50% of the total acceleration in mass loss.

It should be noted that our results are limited to the past two decades (2003–2020) and thus are susceptible to interannual to decadal climate variability. For example, high interannual variability in mass changes, modulated by natural phenomena like El Ninõ Southern Oscillation (ENSO) and the Southern Annular Mode (SAM), are dominant signals in both regional and continental-scale SMB anomalies (5, 50, 51). Extreme snowfall events, partly caused by the occurrence of atmospheric rivers, would also significantly affect the contribution of SMB to ice mass changes (52). While some relatively gradual changes in ice discharge are obviously caused by large-scale (oceanic) processes, such as in the Amundsen and Bellingshausen Sea coast of West Antarctica, some individual glaciers exhibit strong interannual changes, probably as a result of local oceanographic variability (5, 15, 37). Consequently, accurate observation of these short-term dynamics is crucial for understanding of regional contribution to future ice mass balance.

Our results not only provide insight into the intricate imbalance of ice discharge and SMB but also underscore the need for integrating multiple observational datasets and associated numerical modeling to refine our understanding of the AIS ice mass balance. Precise delineation of the rheological properties of the Antarctic upper mantle could refine viscoelastic response to historical and contemporary ice mass unloading, thereby improving ice mass observations from satellite gravimetry and altimetry. Additionally, refining bed topography is crucial for the flux gate method to reduce potential systematic errors, and enhancing the SMB models is key for accurately identifying the drivers of short-term ice mass/volume changes across various observational methods and reducing associated uncertainties (53). With the ongoing impact of Antarctic ice mass loss on global sea levels, such efforts will become crucial for accurately projecting future sea level rise.

## Materials and Methods

### Ice Mass Change.

To estimate Antarctic ice mass change, we used the constrained linear deconvolution (CLD) method developed by ref. 22. This approach spatially combines ice mass changes derived from satellite gravimetry (GRACE and GRACE Follow-On (GRACE-FO)) and multimission satellite altimetry. Compared with previous approaches that combined GRACE and altimetry data (54–56), this method effectively resolves spatial leakages inherently presented in GRACE data and provides higher temporal resolution (monthly intervals). Let the ice mass loads from GRACE and GRACE-FO to be $M_{G}$, and those from altimetry to be $〈m〉$, the combined estimates ($\hat{m}$) can be calculated using a linear deconvolution as follows (22):

[1] $$\begin{array}{c} \hat{m}=〈m〉+{\left[G^{T}PG+\lambda Q\right]}^{-1}G^{T}P\left[M_{G}-G〈m〉\right], \end{array}$$

where G is the Gaussian smoothing operator, and P and Q are diagonal matrices where elements are inverses of GRACE/GRACE-FO and altimetry error, respectively. λ is a regularization parameter, balancing between the prediction error and the solution simplicity. The resulting mass load estimates ($\hat{m}$) achieve high spatial resolution (~27 km) as it includes satellite altimetry observations as an a priori, but the larger scale (~ 300 km) ice mass change is almost identical to GRACE observations as the solution minimizes the difference from GRACE observations ($M_{G}$). This approach greatly reduces signal leakage errors in GRACE data by setting the ocean element of matrix Q to be very large. It also suppresses uncertainties in altimetry observations by adding back the deconvolution of $M_{G}-G〈m〉$ to $〈m〉$ (Eq. **1**). The final solutions from Eq. **1** show similar ice mass variations to estimates from the flux gate method (15) at the glacial scale during 2003–2016 (22).

For $M_{G}$, we used observations from GRACE (2003–2017) and GRACE-FO (2018-) satellites (57, 58). The Release 6 level 2 monthly spherical harmonic solution provided by the Center for Space Research was used for both satellite datasets. The Release 6 solution improves upon the atmospheric pressure correction errors found in previous data version (19, 59), providing more accurate observations of ice mass changes in Antarctica (60). The inaccurate low-degree coefficients were replaced with independent estimates according to GRACE’s Technical Notes #13a and #14 (61–63). We corrected the GIA effect using a model output based on ICE-6G ice history (64), which is widely adopted in recent studies. We successively applied a decorrelation (65) and a 400 km Gaussian filters, converted the filtered spherical harmonic coefficients into surface mass loads at 27 km intervals on the polar-stereographic coordinates. We removed the ocean mass signals due to sea level fingerprint (66) using leakage-corrected land mass signals (67) as performed in ref. 22.

The estimated mass loads were further modified considering the uncertainty of the GIA model used here. A recent study (20) reported that the GIA model used here (64) underestimates the uplift signals near the glaciers in Amundsen Sea Embayment (ASE) when compared with observations from the GNSS network. The authors suggested that the mass loss trend signals from GRACE observation corrected by the same model should be inflated by 10% in the region. This suggestion further has been supported by (22), finding that the 10% inflation in GRACE’s signal further increases the agreement in glacial-scale mass variations between the CLD and the flux gate method estimates. Therefore, we inflated the mass loss trends in ASE signals with 10% scaling of total (as in ref. 22), applying this modification in $M_{G}$ of Eq. **1**.

For $〈m〉$, we used multimission satellite altimetry data estimated by ref. 68, which provides monthly surface elevations at approximately 2 km grid intervals from 1985 to 2020. We used data from 2003 to 2020, corresponding to GRACE/GRACE-FO data period, including mission periods of Envisat (2002–2012), ICESat (2003–2009), CryoSat-2 (2010-), and ICESat-2 (2018-). Missing grid points were filled through space-time interpolation and extrapolation (68). We converted volume to mass following ref. 22 using a firn densification (69) and SMB model outputs (30). Finally, we resampled the estimated mass changes to 27 km grid intervals and matched the time intervals of GRACE data. The time series of total AIS mass change from $〈m〉$, along with our final estimates ($\hat{m}$), is presented in *SI Appendix*, Fig. S1.

For uncertainties of GRACE/GRACE-FO included in matrix P, we used formal uncertainty of GRACE and GRACE-FO data. In the case of altimetry uncertainties for Q, we used the error from ref. 68 estimated from the analysis of cross-over observations.

### SMB.

For mass balance partitioning, for SMB, we used output of the Regional Atmospheric Climate Model (RACMO) version 2.3p2 (30). RACMO is forced by European Centre for Medium-Range Weather Forecasts (ECWMF) reanalysis, which is known for its accurate representation of SMB variability over Antarctica (31). The data provide SMB variables with a relatively high spatial resolution (27 km) at monthly time intervals since January 1979. A 10% uncertainty in monthly SMB was assumed based on the SMB bias estimates against in situ observations (30).

### Ice Mass Change Partitioning.

The ice mass changes over a glacier basin (ΔM) represents/equals the difference between the time-integrated monthly SMB (SMB) and ice discharge (D):

[2] $$\Delta M\left(t\right)=\int SMB\left(t\right)\;dt-\int D\left(t\right)\;dt.$$

If time-integrated SMB and D are equal over a given period, ΔM will be zero. Any anomalous variations in these two components will create an imbalance that causes an increase or decrease in ΔM. These anomalous variations can also be determined by comparing the values to their long-term means ($SMB_{\mathrm{ref}}$ and $D_{\mathrm{ref}}$). Assuming that the long-term SMB is balanced by ice discharge ($SMB_{\mathrm{ref}}$ =$D_{\mathrm{ref}}$), we can modify the right-hand side of Eq. (**2**) as follows:

[3] $$\begin{array}{c} \Delta M\left(t\right)=\int \left(SMB\left(t\right)-SMB_{\mathrm{ref}}\right)\;dt-\int \left(D\left(t\right)-D_{\mathrm{ref}}\right)\;dt=\Delta SMB\left(t\right)-\Delta D\left(t\right), \end{array}$$

where ΔSMB and ΔD are anomalous components of the cumulative SMB and ice discharge, respectively, and the reference period is considered as 30 y from 1979 to 2008 in line with recent studies (15, 27–29). This implicitly assumes that the SMB and D are balanced during the reference period (i.e., $SMB_{\mathrm{ref}}$ = $D_{\mathrm{ref}}$). For straightforward comparison in the mass balance partitioning, the negative sign of ΔD (-ΔD) is used.

In the flux gate method, D is estimated by multiplying the ice speeds from satellite imagery with the ice thickness at the grounding line from multisource data (15). The difference between ΔSMB and ΔD (Eq. **3**) provides an indirect estimate of ΔM. However, in this study, ΔM is directly estimated from CLD (=$\int_{S}\hat{m}\;dS$, where S is the spatial domain of a given basin), and ΔD is estimated indirectly by subtracting ΔSMB from ΔM.

Eq. **3** was used for all basins except the basin including the Kamb Ice Stream in West Antarctica (in basin 18). The ice flow at the trunk of Kamb Ice Stream is known to have stagnated about 160 y ago (70, 71), and there is no present-day ice discharge at the grounding line (D =0). This situation allows upstream ice flow accumulated in the middle of the ice stream basin and generates local increasing mass trends (22). As a result, the total ice mass in this area is controlled only by the SMB accumulations due to the absence of ice discharge at the groundling line, which makes the $D_{\mathrm{ref}}$ = $SMB_{\mathrm{ref}}$ assumption invalid in this case (i.e., $D_{\mathrm{ref}}$ =0). Therefore, for this region, ΔM was simply compared with ∫SMB according to Eq. **2**.

From ΔM and ΔSMB, we estimated linear trends and accelerations, which are critical parameters for representing long-term variability. These two parameters were estimated by a regression function that includes annual and semiannual components:

[4] $$\begin{array}{c} f\left(t\right)=a_{0}+a_{1}t+\frac{1}{2}a_{2}t^{2}+a_{3}\sin\left(2\pi t\right)+a_{4}\cos\left(2\pi t\right)+a_{5}sin\left(4\pi t\right)\\ \;+a_{6}cos\left(4\pi t\right), \end{array}$$

where $a_{1}$ and $a_{2}$ are linear trends and accelerations to be estimated. In practice, the regression was performed using Hector software (72) to account for the correlated characteristics of errors (see next section for more details).

### Uncertainties.

To comprehensively evaluate the statistical significance of the estimated linear trend and accelerations in ΔM, we considered three different sources of error.

The first source is a random error, which arises in the estimation of $\hat{m}$. We estimated this error using Monte Carlo (MC) simulation, generating 10,000 set of random numbers corresponding to the uncertainties in GRACE/GRACE-FO and altimetry. Each set of random numbers (i.e., expected uncertainty) associated with GRACE/GRACE-FO and altimetry were inputted into $M_{G}$ and $〈m〉$ in Eq. **1** respectively, and $\hat{m}$ was calculated as the uncertainty (22). From these simulated time series, we estimated linear trends and accelerations based on Eq. **4** and considered their two SD as the random errors.

The second source of error is regression error, caused by the residuals between the mass change time series and the regression model. We used Hector software (72) to estimate this error, which yields regression parameters and their uncertainties considering the correlated characteristics of the residual noises (73). The regression model was established according to Eq. **4**, and the Generalized Gaussian Markov model was adopted as the optimal noise model (74).

The systematic error, which predominantly affects linear trends, is primarily caused by inaccuracies in the GIA model corrected for GRACE data (22). We assumed this error to be the maximum difference in trends between several GIA models (64, 75–77), similar to previous studies (22, 28).

The total uncertainty in linear trends of ΔM was calculated as the root-sum-square of these three error factors, under the assumption that they are not correlated with each other. The uncertainty of accelerations was estimated as the root-sum-square of random and regression error.

We employed the same sources of error to estimate uncertainties in ΔSMB. For random errors, we assumed the error of SMB to be 10% of monthly SMB and evaluated the uncertainty of its time-integration (ΔSMB) by calculating the root-sum-square of the errors from the first month to the given month ($\sqrt{\sum_{k=1}^{n}\varepsilon_{k}}$, where $\varepsilon_{k}$ is the error of k th month, and n is the index of the given month) (27, 29). The uncertainties of linear trends and accelerations were then estimated by MC simulation with the generated ΔSMB errors. For regression errors, we used Hector software as before. The systematic error in ΔSMB is due to inaccuracy in $SMB_{\mathrm{ref}}$. We considered 10% of annual SMB during the reference period (1979–2008) as SMB errors and assumed their SEM ($\frac{\sigma }{\sqrt{n}}$, in which n is a number of years, and σ is SD) as the systematic error. Finally, the total uncertainty of ΔSMB was estimated as the root-sum-square of these three error factors.

Since -ΔD is estimated indirectly from the difference between ΔM and ΔSMB, its uncertainty would include both sources of uncertainty. We estimated the uncertainty of -ΔD to be the root-sum-square of uncertainties in ΔM and ΔSMB.

Tables S1 and S2 represent the three uncertainty components in linear trends and accelerations, respectively. For all variables, systematic errors exhibit the largest uncertainties in linear trends. For example, in the estimates of entire AIS, the inaccuracy of the GIA model accounts for about 89% (19.3 Gt y^−1^) of uncertainty in ΔM, whereas the uncertainty in the reference SMB ($SMB_{\mathrm{ref}}$) accounts for about 95% (36.3 Gt y^−1^) of uncertainty in ΔSMB. In terms of acceleration, the regression error accounts for more uncertainty than the random error, contributing to about 99% (3.8 Gt y^−2^) of ΔM and about 83% (3.8 Gt y^−2^) of ΔSMB, respectively. The main uncertainty factors in ΔM and ΔSMB also affect the uncertainties in -ΔD.

## Supplementary Material

Appendix 01 (PDF)

Dataset S01 (XLSX)

## Data Availability

The time series of ΔM and ΔSMB (which can also be used to calculate -ΔD) for the basins and sub-basins estimated in this study are provided in *Dataset S1*. The high-resolution gridded ice mass change estimates are openly available via the Korea Polar Data Center (KPDC, https://dx.doi.org/doi:10.22663/KOPRI-KPDC-00002466.1) (78).
