# Supplementary material for: Partitioning the drivers of Antarctic glacier mass balance (2003–2020) using satellite observations and a regional climate model
Source: Proc Natl Acad Sci U S A. 2024 Sep 30;121(41):e2322622121. doi: 10.1073/pnas.2322622121 (PMC11474090; doi:10.1073/pnas.2322622121)
Supplement: Supplementary file 1 — Appendix 01 (PDF) [file pnas.2322622121.sapp.pdf]

PNAS

Supporting Information for

Partitioning the drivers of Antarctic glaciers mass balance (2003-2020) using satellite observations and a regional climate model

Byeong-Hoon Kim[1], Ki-Weon Seo[2*], Choon-Ki Lee[1], Jae-Seung Kim[2], Won Sang Lee[1], Emilia Kyung Jin[1], and Michiel van den Broeke[3]

[1]Division of Glacier & Earth Sciences, Korea Polar Research Institute, Incheon, Republic of Korea
[2]Department of Earth Science Education, Seoul National University, Seoul, Republic of Korea
[3]Institute for Marine and Atmospheric Research, Utrecht University, Utrecht, The Netherlands

*Ki-Weon Seo
Email: seokiweon@snu.ac.kr

This PDF file includes:

Figures S1 to S10
Table S1 to S3
SI References

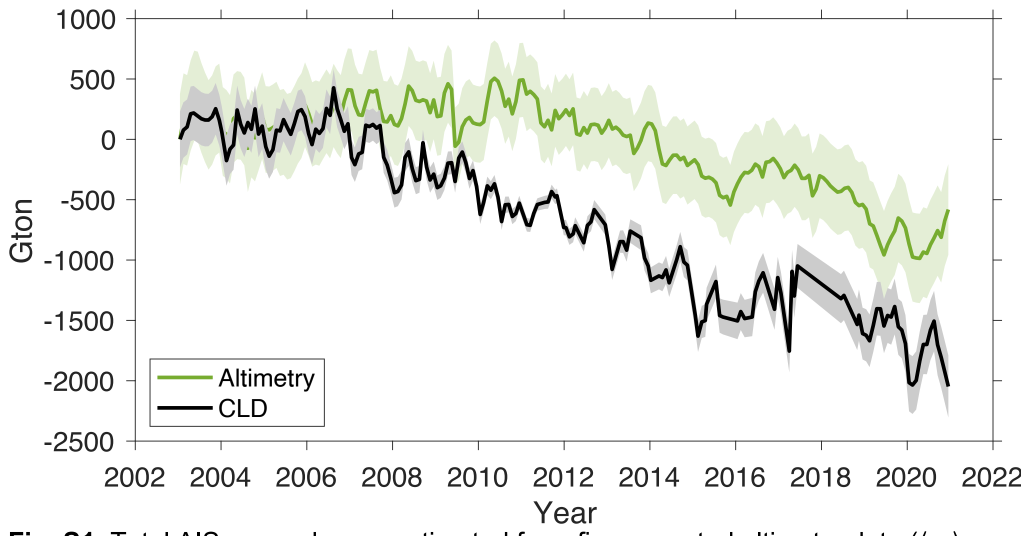

Fig. S1. Total AIS mass change estimated from firn-corrected altimetry data ($\langle \boldsymbol{m} \rangle$, green) and the CLD method ($\hat{\boldsymbol{m}}$, black).

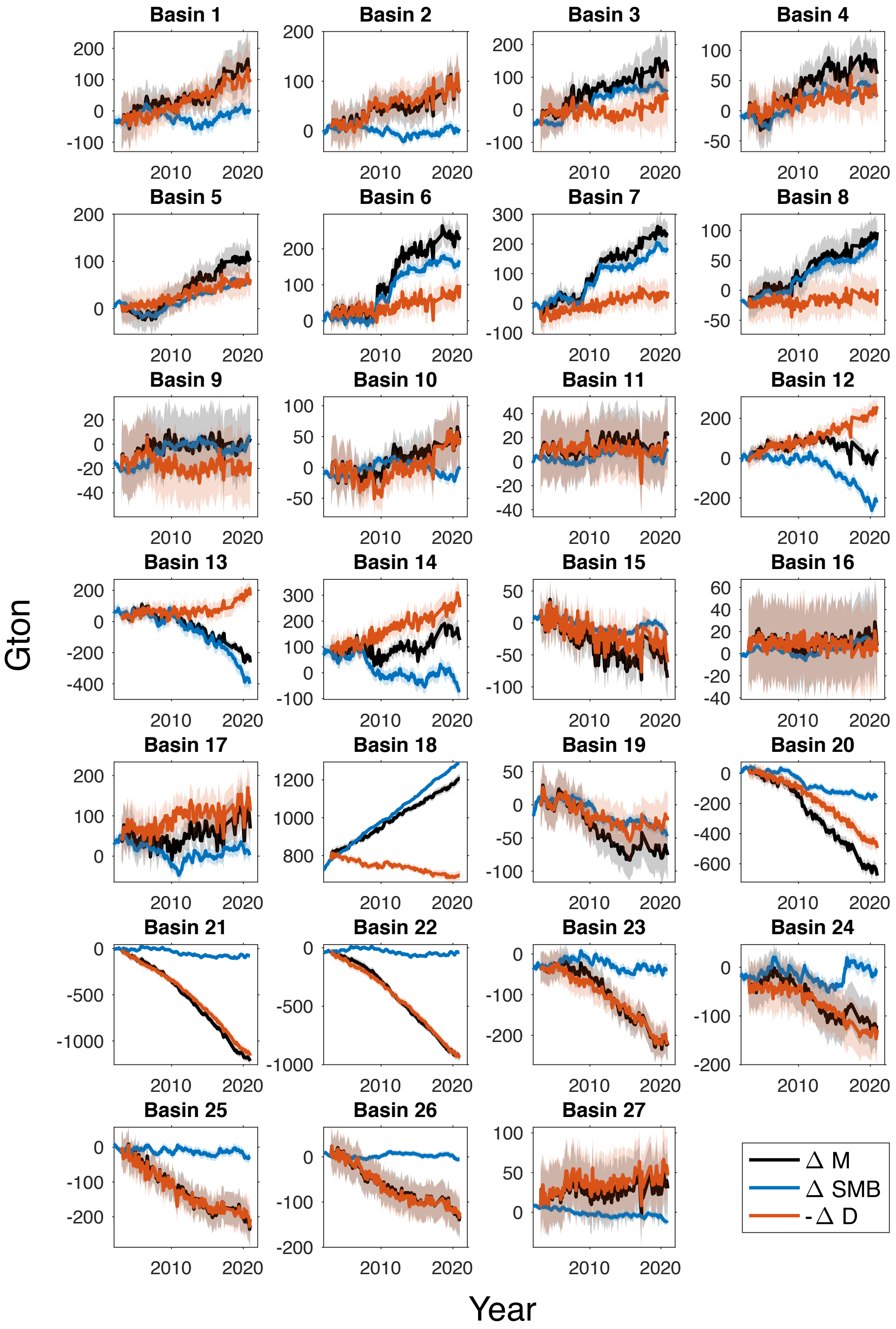

Fig. S2. ΔM (black), ΔSMB (blue), and $-\Delta D$ (red) for 27 drainage basins.

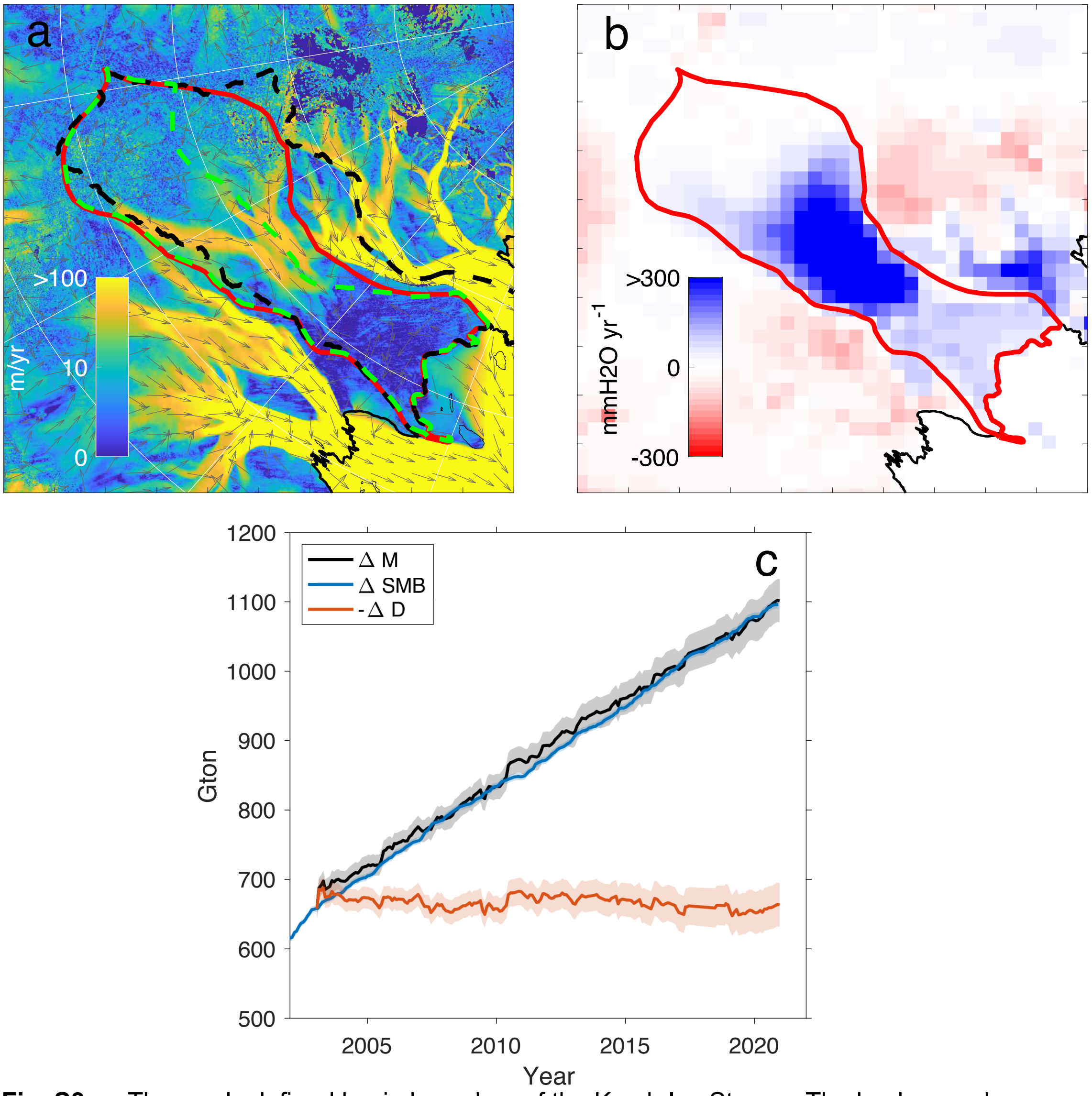

Fig. S3. a: The newly defined basin boundary of the Kamb Ice Stream. The background map represents the ice velocity field from MEaSUREs2 project (1), with gray arrows indicating the ice flow directions. The black dashed line represents the boundary estimated by surface altitude observations from ICESat altimetry (2), while the green dashed line represents the boundary from ice speed observations from satellite imagery (3). We redefined the basin boundary based on the velocity vectors (gray arrows) converging toward the trunk of the Kamb Ice Stream, as indicated by the red line. b: A map of ice mass trend overlaid with the new basin boundary. c: Ice mass changes partitioning calculated from the new basin boundary. Note that each graph is vertically adjusted to enhance visualization.

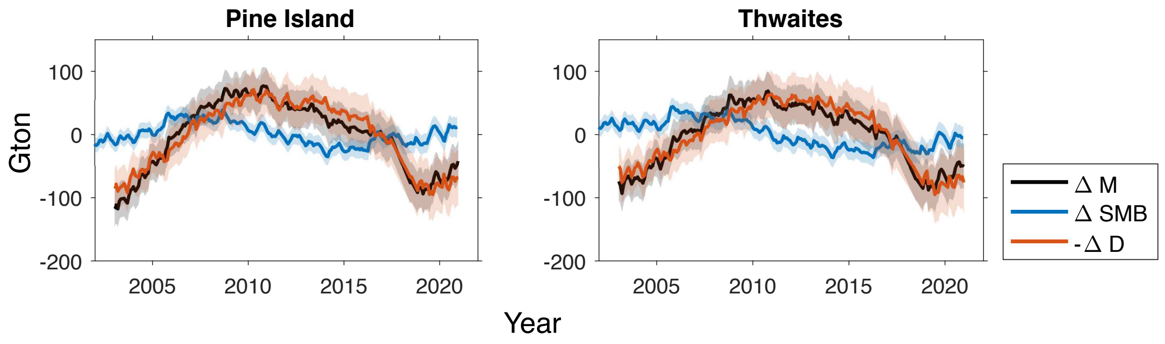

Fig. S4. Detrended mass changes over Pine Island (left) and Thwaites Glaciers (right).

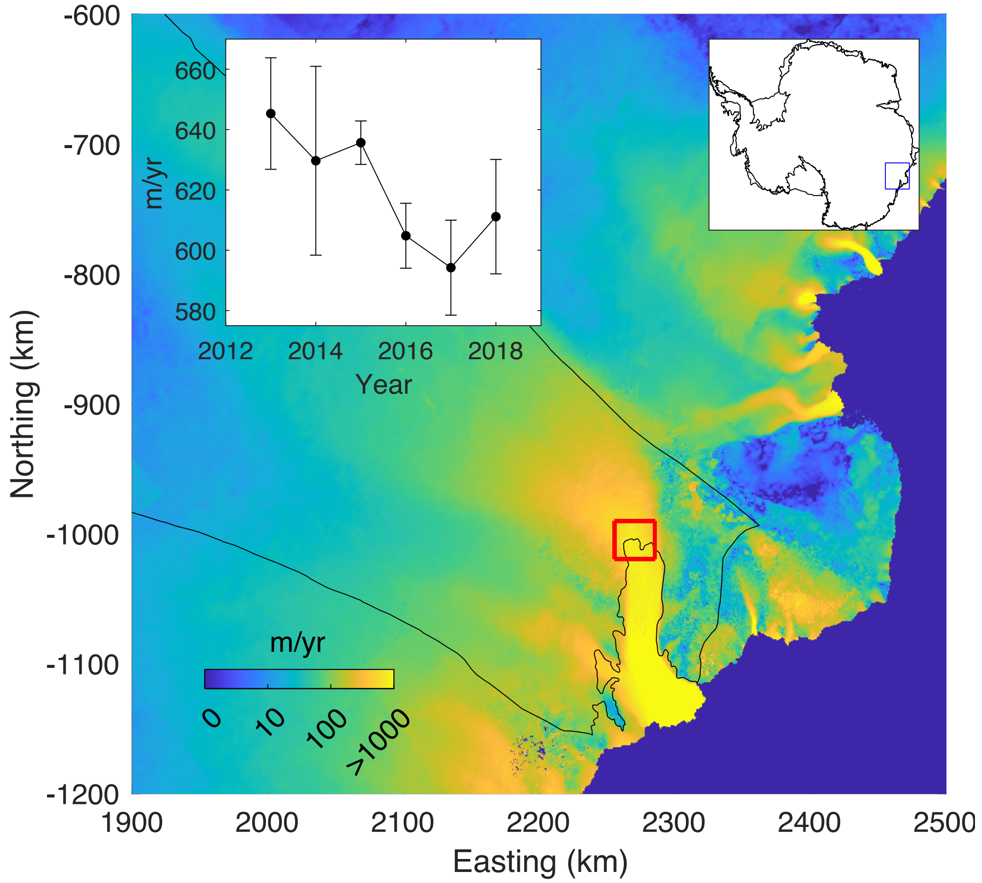

Fig. S5. Ice velocity changes near the grounding line of the Totten Glacier (top-left inset). The background image shows the ice velocity filed in 2018, and the red box indicates the region used for calculating the velocity time series. The top-right inset shows the location of the background image. We used velocity data generated using auto-RIFT (4) and provided by the NASA MEaSUREs ITS_LIVE project (5).

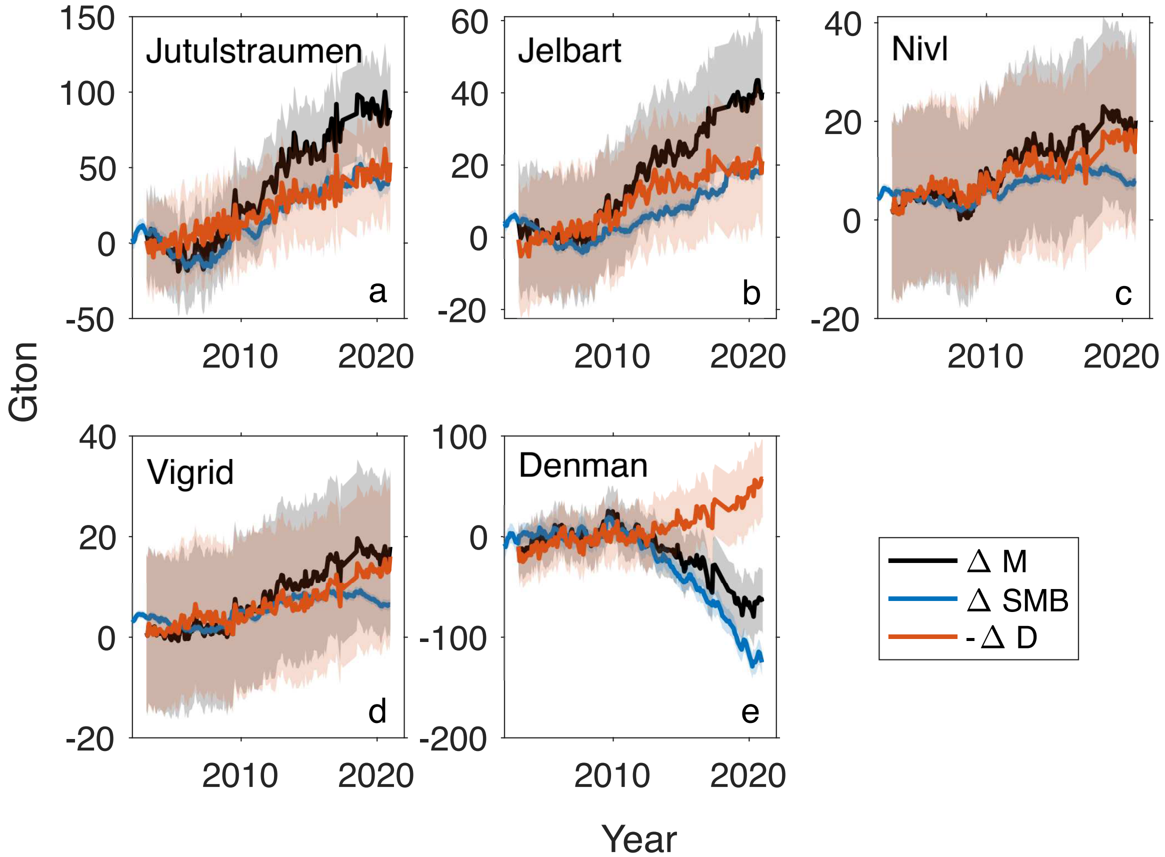

Fig. S6. $\Delta\boldsymbol{M}$ (black), $\Delta\boldsymbol{SMB}$ (blue), and $-\Delta\boldsymbol{D}$ (red) for Jutulstraumen, Jelbart, Nivl, Vigrid, and Denman Glaciers.

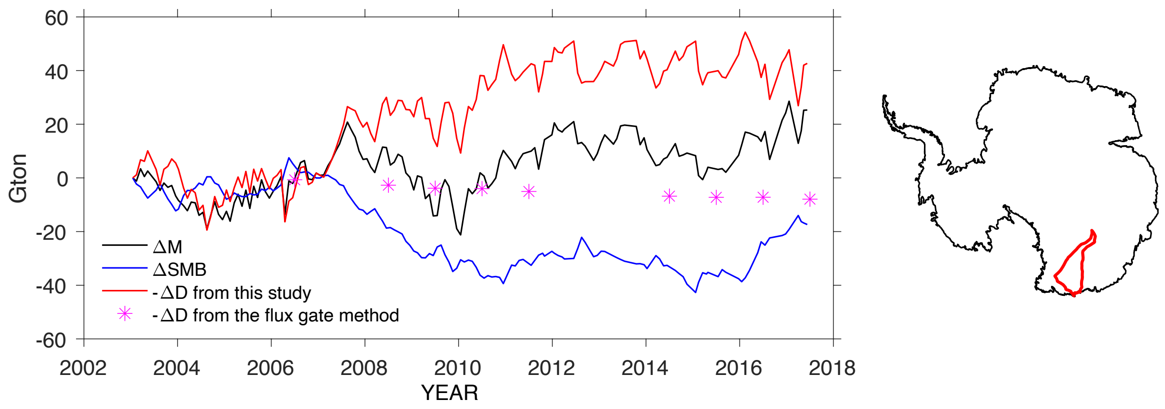

Fig. S7. Comparison of $-\Delta\boldsymbol{D}$ time series estimated by this study (red line) and those from the flux gate method (magenta stars, ref. (6)) at glaciers flowing into Cook Ice Shelf (outlined in red on the right panel). For comparison, $\Delta\boldsymbol{M}$ (black line) and $\Delta\boldsymbol{SMB}$ (blue line) are shown together.

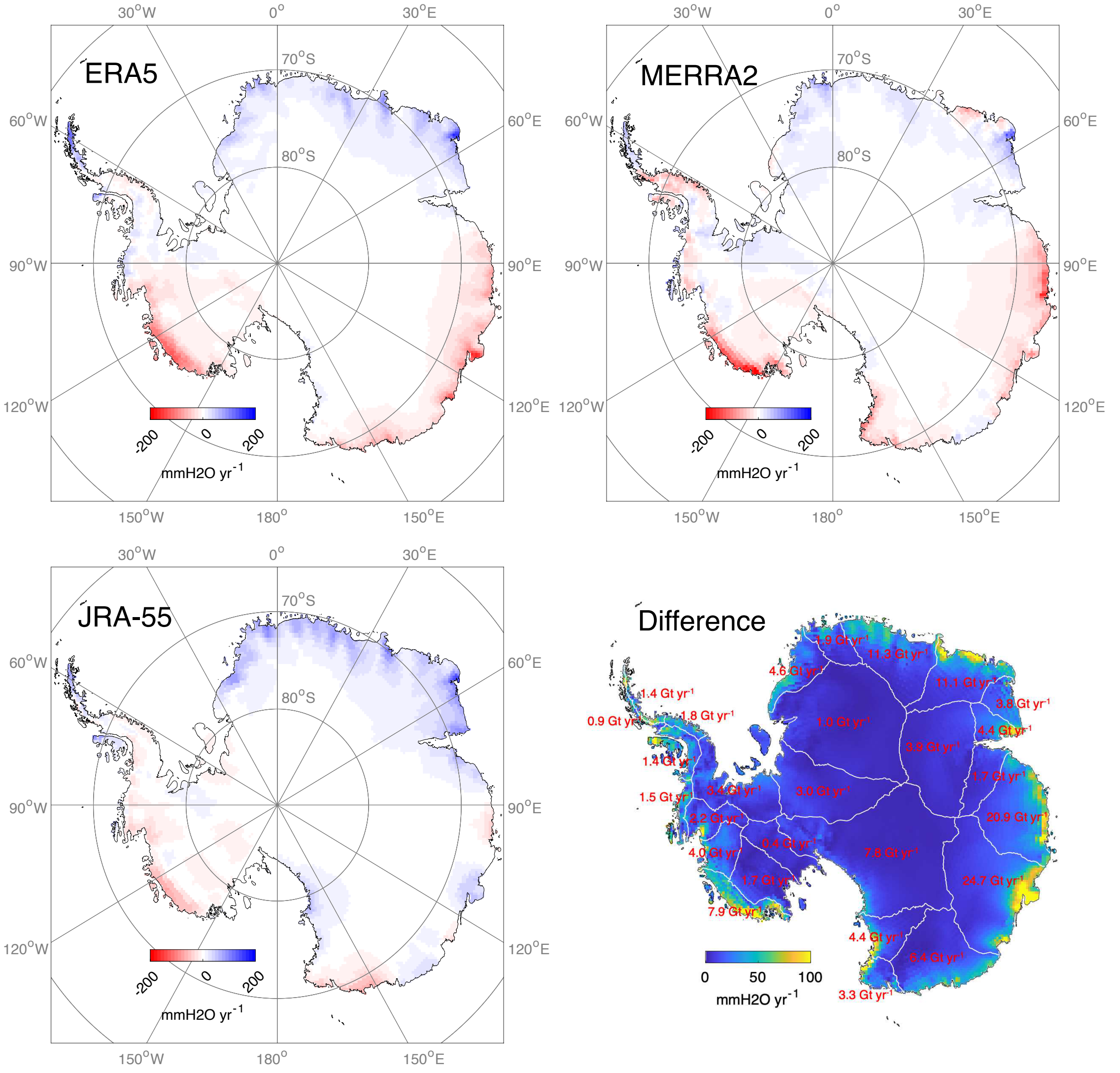

Fig. S8. Linear trends of Antarctic $\Delta\boldsymbol{SMB}$ derived from three reanalysis models (ERA5, MERRA2, JRA-55) during 2003-2020. Note that for MERRA2, the climatology period for calculating $\boldsymbol{SMB_{ref}}$ is restricted to 1980-2008 due to data availability, whereas for ERA5 and JRA-55, it is 1979-2008. The bottom right panel shows the maximum deviation among the linear trends of each model, with the maximum deviations for basin-integrated values also indicated.

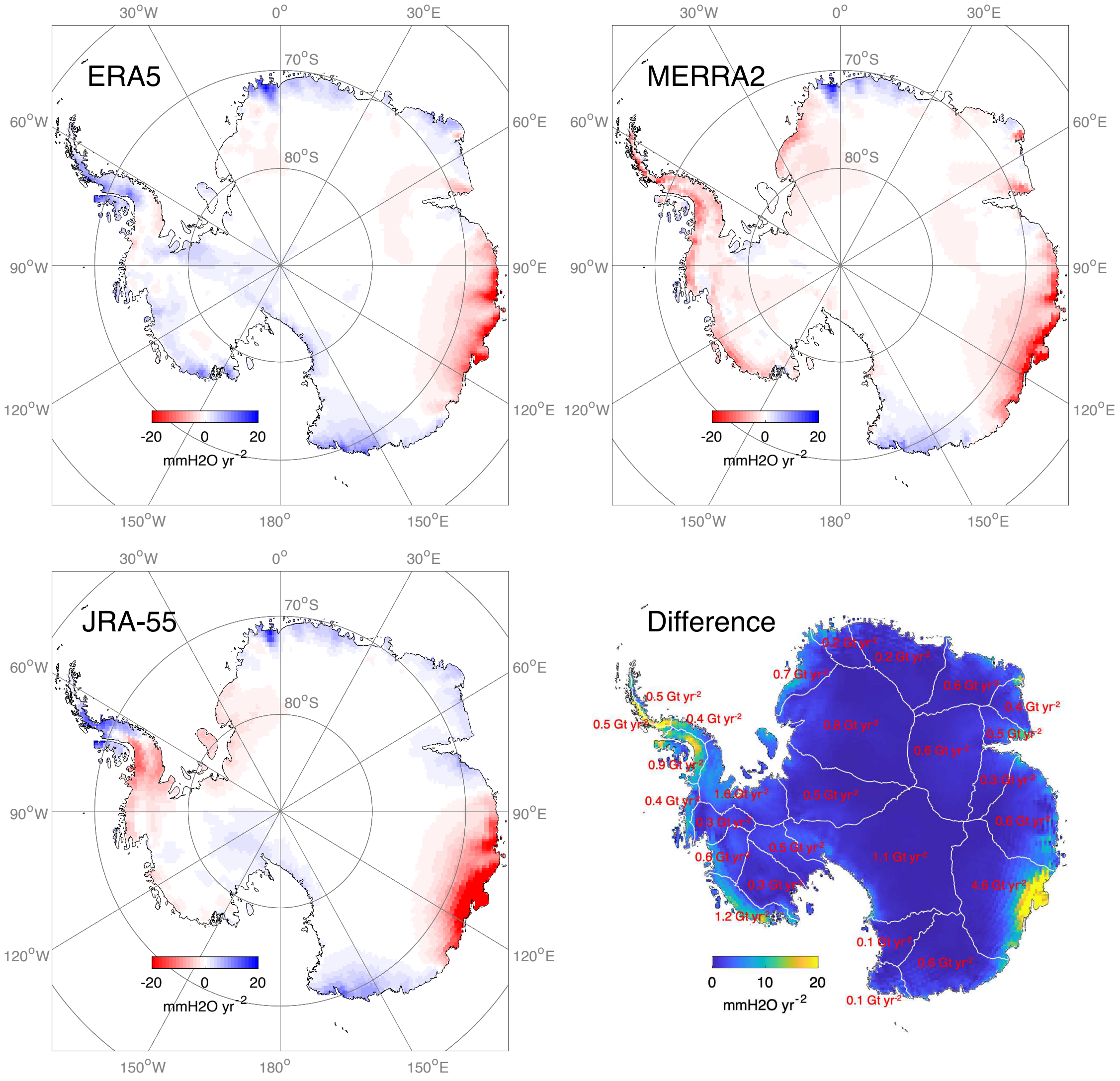

Fig. S9. Similar to Fig. S8 except for acceleration components.

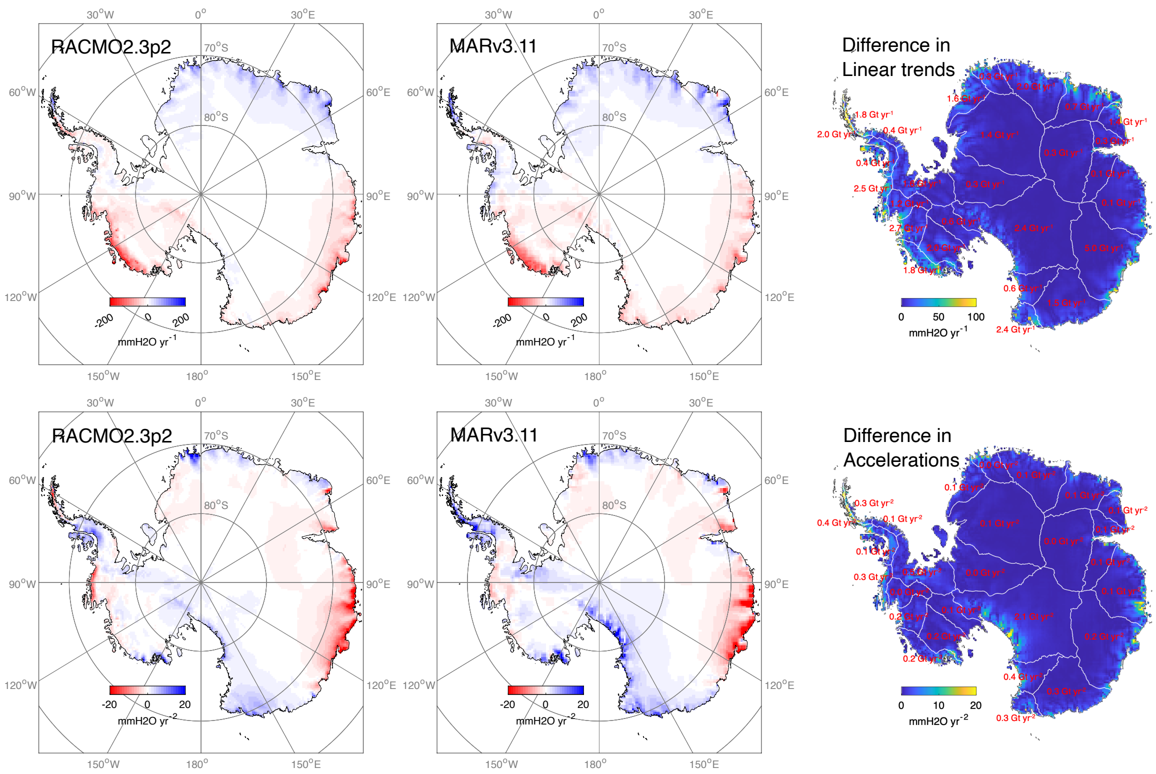

Fig. S10. Linear trends (top) and acceleration (bottom) of Antarctic ΔSMB suggested by two regional climate models (RACMO and MAR). The deviations between the models are shown on the right. Note that this comparison was performed over a shorter period (2003-2019) compared to the main text (2003-2020) due to the data availability of MAR.

Table S1. Three uncertainty components of linear trends (unit = Gton yr^{-1}). Total uncertainties are root-sum-square of the three components, which is the same as presented in Table 1.

Basin number	$\Delta \boldsymbol{M}$				$\Delta \boldsymbol{SMB}$				$\Delta \boldsymbol{D}$
	Regression error	Random error	Systematic (GIA) error	Total	Regression error	Random error	Systematic (SMB_{ref}) error	Total	
1	1.8	1.1	8.5	8.7	1.9	0.6	2.3	3.1	9.3
2	0.9	0.9	3.2	3.4	0.6	0.3	1.0	1.1	3.6
3	0.9	0.8	9.2	9.3	1.1	0.4	1.4	1.8	9.4
4	1.2	0.8	2.8	3.2	1.1	0.3	0.9	1.4	3.5
5	1.6	0.8	0.7	1.9	1.4	0.2	0.7	1.6	2.5
6	3.5	0.8	2.0	4.1	4.0	0.4	1.4	4.2	5.9
7	2.4	0.8	3.4	4.3	2.2	0.5	1.5	2.7	5.1
8	0.7	0.8	1.2	1.6	0.7	0.2	0.6	1.0	1.9
9	0.4	0.7	1.0	1.3	0.3	0.1	0.4	0.5	1.4
10	0.9	0.7	4.5	4.7	0.4	0.3	0.9	1.0	4.8
11	0.4	0.7	1.6	1.8	0.3	0.1	0.3	0.5	1.8
12	1.2	0.8	3.3	3.6	1.5	0.8	2.9	3.4	5.0
13	1.8	0.7	1.1	2.3	2.1	1.3	4.6	5.3	5.7
14	2.2	0.8	3.1	3.9	2.3	0.8	2.8	3.7	5.4
15	0.6	0.8	1.3	1.6	0.5	0.2	0.6	0.8	1.8
16	0.2	1.0	0.9	1.4	0.3	0.1	0.2	0.4	1.4
17	1.1	1.0	5.1	5.3	1.2	0.5	1.7	2.1	5.8
18	0.4	0.5	1.8	1.9	0.4	0.2	0.0	0.5	2.0
19	1.3	0.8	2.5	2.9	0.8	0.3	0.9	1.2	3.2
20	3.1	1.1	1.6	3.6	2.2	0.7	2.4	3.3	4.9
21	2.7	1.0	2.1	3.6	1.8	0.6	2.1	2.8	4.5
22	3.7	0.8	1.0	3.9	2.1	0.4	1.6	2.6	4.7
23	1.5	0.9	1.8	2.5	0.9	0.4	1.3	1.6	3.0
24	1.9	1.3	1.0	2.5	1.7	0.4	1.5	2.3	3.4
25	0.6	1.2	2.9	3.2	0.7	0.3	1.3	1.5	3.6
26	0.6	1.1	1.7	2.1	0.4	0.2	0.7	0.8	2.2
27	0.6	1.1	1.0	1.6	0.2	0.1	0.4	0.5	1.7
West	13.1	1.5	14.5	19.6	8.9	2.9	11.0	14.4	24.3
East	9.5	1.6	23.6	25.5	10.6	5.6	21.6	24.7	35.5
Peninsula	3.5	1.4	4.4	5.8	2.4	1.0	3.8	4.6	7.4
Antarctica	9.4	2.3	19.3	21.6	7.1	9.4	36.3	38.2	43.9

Table S2. Two uncertainty components of accelerations (unit = Gton yr^{-2}). Total uncertainties are root-sum-square of the two and is the same with those presented in Table 1.

Basin number	ΔM			ΔSMB			ΔD
	Regression error	Random error	Total	Regression error	Random error	Total	
1	0.7	0.2	0.7	0.7	0.1	0.8	1.0
2	0.4	0.2	0.4	0.2	0.1	0.2	0.5
3	0.4	0.2	0.4	0.4	0.1	0.4	0.6
4	0.5	0.2	0.5	0.4	0.1	0.4	0.7
5	0.5	0.2	0.6	0.5	0.0	0.5	0.7
6	1.2	0.2	1.2	1.4	0.1	1.4	1.9
7	0.9	0.2	0.9	0.8	0.1	0.8	1.2
8	0.3	0.2	0.3	0.3	0.0	0.3	0.4
9	0.1	0.2	0.2	0.1	0.0	0.1	0.2
10	0.3	0.1	0.4	0.2	0.1	0.2	0.4
11	0.2	0.1	0.2	0.1	0.0	0.1	0.3
12	0.5	0.2	0.5	0.6	0.2	0.6	0.8
13	0.8	0.2	0.8	0.8	0.3	0.9	1.2
14	0.9	0.2	0.9	0.9	0.2	0.9	1.3
15	0.2	0.2	0.3	0.2	0.0	0.2	0.4
16	0.1	0.2	0.2	0.1	0.0	0.1	0.3
17	0.5	0.2	0.5	0.5	0.1	0.5	0.7
18	0.2	0.1	0.2	0.2	0.0	0.2	0.3
19	0.5	0.2	0.5	0.3	0.1	0.3	0.6
20	1.2	0.2	1.2	0.8	0.1	0.9	1.5
21	1.0	0.2	1.0	0.7	0.1	0.7	1.2
22	1.1	0.2	1.1	0.8	0.1	0.8	1.4
23	0.6	0.2	0.6	0.4	0.1	0.4	0.7
24	0.7	0.3	0.8	0.7	0.1	0.7	1.0
25	0.3	0.2	0.4	0.3	0.1	0.3	0.5
26	0.2	0.2	0.3	0.2	0.0	0.2	0.4
27	0.2	0.2	0.3	0.1	0.0	0.1	0.3
West	4.4	0.3	4.4	3.5	0.6	3.5	5.6
East	3.7	0.3	3.7	4.0	1.2	4.2	5.6
Peninsula	1.3	0.3	1.4	0.9	0.2	1.0	1.7
Antarctica	3.8	0.5	3.8	2.9	2.0	3.5	5.2

Table S3. Revised list of glacier basins for comparative analysis presented in Fig. 3. The name of the glacier basins are consistent with those used by ref. (6).

Trends				Accelerations			
**No.**	**Name**	**Basin**	**Area (km^2)**	**No.**	**Name**	**Basin**	**Area (km^2)**
1	Carlson	West	22,616	1	Binchadler	West	127,034
2	Echelmeyer	West	19,405	2	Carlson	West	22,616
3	Evans	West	124,091	3	Evans	West	124,091
4	MacAyeal	West	191,796	4	Institute	West	160,429
5	Rutford	West	58,231	5	Abbot	West	26,738
6	Crosson	West	12,775	6	Crosson	West	12,775
7	Dotson	West	17,429	7	Dotson	West	17,429
8	Ferringo	West	9,394	8	Ferringo	West	9,394
9	Fox	West	4,055	9	Fox	West	4,055
10	Getz	West	85,913	10	Getz	West	85,913
11	Haynes	West	9,812	11	Haynes	West	9,812
12	Hull	West	16,894	12	Hull	West	16,894
13	Land	West	13,266	13	Pine Island	West	181,421
14	Pine Island	West	181,421	14	Thwaites	West	192,759
15	Sulzberger	West	39,416	15	Vernable	West	14,901
16	Thwaites	West	192,759	16	Byrd	East	933,744
17	Vernable	West	14,901	17	Mellor	East	439,736
18	Academy	East	411,989	18	Mulock	East	136,582
19	Byrd	East	933,744	19	Nimrod	East	283,664
20	Nimrod	East	283,664	20	Slessor	East	493,307
21	Cook	East	308,184	21	Cook	East	308,184
22	David	East	213,553	22	David	East	213,553
23	Denman	East	265,483	23	Denman	East	265,483
24	Dibble	East	32,169	24	Dibble	East	32,169
25	Frost	East	155,082	25	Totten	East	556,081
26	Jelbart	East	19,965	26	George VI	Peninsula	80,174
27	Jutulstraumen	East	190,916	27	Larsen C	Peninsula	18,115
28	Matusevitch	East	17,394	28	Stange	Peninsula	15,008
29	Mertz	East	84,600	29	West Graham Land	Peninsula	25,156
30	Moscow	East	211,595				
31	Ninis	East	178,748				
32	Nivl	East	27,656				
33	Rennick	East	52,383				
34	Totten	East	556,081				
35	Vigrid	East	35,700				
36	Vincennes Bay	East	134,559				
37	Flemming	Peninsula	5,742				
38	George VI	Peninsula	80,174				
39	Stange	Peninsula	15,008				
40	West Graham Land	Peninsula	25,156				

References

1. E. Rignot, J. Mouginot, B. Scheuchl, MEaSUREs InSAR-Based Antarctica Ice Velocity Map. Boulder, Colorado USA: NASA DAAC at the National Snow and Ice Data Center. 10.5067/MEASURES/CRYOSPHERE/nsidc-0484.001 (2011).
2. H. J. Zwally, B. G. Mario, A. B. Matthew, L. S. Jack, Antarctic and Greenland Drainage System, GSFC Cryospheric Sciences Laboratory, at http://icesat4.gsfc.nasa.gov/cryo_data/ant_grn_drainage_systems.php. (2012).
3. J. Mouginot, B. Scheuchl, E. Rignot, MEaSUREs Antarctic Boundaries for IPY 2007-2009 from Satellite Radar, Version 2.
4. A. S. Gardner *et al.*, Increased West Antarctic and unchanged East Antarctic ice discharge over the last 7 years. *The Cryosphere* **12**, 521-547 (2018).
5. A. S. Gardner, M. A. Fahnestock, T. A. Scambos, ITS_LIVE Regional Glacier and Ice Sheet Surface Velocities: Version 1. Data archived at National Snow and Ice Data Center. 10.5067/6II6VW8LLWJ7 (2023).
6. E. Rignot *et al.*, Four decades of Antarctic Ice Sheet mass balance from 1979-2017. *Proc Natl Acad Sci U S A* **116**, 1095-1103 (2019).
